# Potential Value of Matrix Metalloproteinase-13 as a Biomarker for Osteoarthritis

**DOI:** 10.3389/fsurg.2021.750047

**Published:** 2021-10-29

**Authors:** Xing Xin, Qizhao Tan, Fang Li, Zhongqiang Chen, Ke Zhang, Feng Li, Bin Yang, Zhili Xing, Fang Zhou, Yun Tian, Yang Lv, Tengjiao Zhu

**Affiliations:** ^1^International Hospital, Third Hospital, Peking University, Beijing, China; ^2^Engineering Research Center of Bone and Joint Precision Medicine, Ministry of Education, Beijing, China

**Keywords:** osteoarthritis, matrix metalloproteinase-13, biomarker, enzyme-linked immunosorbent assay, association

## Abstract

**Background:** Emerging knowledge has highlighted the role of matrix metalloproteinase (MMP)-13 in osteoarthritis (OA); however, the suitability of MMP-13 as a biomarker for OA remains unclear. Therefore, this study aimed to assess the potential value of MMP-13 as a biomarker for OA.

**Methods:** The study enrolled 51 patients, of which 33 had advanced varus OA and 18 did not have OA. Immunohistochemistry and western blotting analyses were performed to measure MMP-13 activity in the cartilage and subchondral bone of patients with OA. Enzyme-linked immunosorbent assay was used to measure serum MMP-13 levels in patients with or without OA. The Western Ontario and McMaster Universities Osteoarthritis Index (WOMAC) was used to assess the association between serum MMP-13 levels and clinical symptoms. Furthermore, the association between serum MMP-13 levels and radiological severity of OA was evaluated using the Kellgren–Lawrence (KL) grading system. Finally, we built the proportional odds logistic regression models to evaluate serum MMP-13 levels as a potential predictor for OA.

**Results:** MMP-13 levels were significantly higher in the severe-worn cartilage of the medial tibial plateau than in the relatively intact portion of the lateral cartilage (*p* < 0.05). This was contrary to the findings for MMP-13 differential expression in the subchondral bone in knee OA (*p* < 0.05). Patients with OA had significantly higher serum MMP-13 levels compared with patients without OA. Additionally, remarkable associations among serum MMP-13 levels, WOMAC scores, and KL grading scores were found in the end-stage OA. Furthermore, the subsequent analysis suggested that serum MMP-13 level was a significant predictor for OA.

**Conclusion:** MMP-13 is valuable for diagnosing, measuring disease severity, and predicting OA in the advanced period of the disease, suggesting that it has potential possibility as a biomarker for OA. However, the underlying mechanisms and clinical application of MMP-13 as a biomarker for OA require to be further investigated.

## Introduction

Osteoarthritis (OA) reportedly affects almost 250 million people worldwide (1), with the knee joint as the most involved site (2). Joint failure and dysfunction in the late stage of knee OA pose substantial health and economic challenges for individuals and society (1, 3, 4). However, the mechanisms of the initiation and progression of knee OA remain are not fully understood (5). The diagnosis of OA depends on classical presentation and radiography findings, which often reveal OA at the end stage when patients often require surgical intervention. It has been reported that molecular changes appear much earlier than apparent clinical symptoms (6). Hence, searching of biomarkers for OA has become a popular research trend (7). However, up to now, to the best of our knowledge, there have been no clinically applicable biomarkers in favor of the diagnosis, monitoring, and assessment the severity of OA owing to the heterogeneity in the pathogenesis and multiformity of clinical symptoms (8).

The pathological breakdown of type II collagen and aggrecan (9, 10) is an important event in knee OA. MMP-13, an important zinc-dependent endopeptidase with preferential activity toward collagen II and aggrecan (11), is involved in this process. Several studies have confirmed its important roles in the osteoarthritic process of the knee joint (12–14). Up to date, there are evidences for the involvement of MMP-13 in many diseases. It has been demonstrated that MMP-13 has a strong relation with diffusion-weighted image lesion increase in human stroke (15). Additionally, a previous study showed that MMP-13 may serve as an independent prognostic factor for invasive breast cancer patients (16). Moreover, a selective MMP-13 inhibitor had been demonstrated to have protective effects on cartilage in a dog OA model (17). These findings support that MMP-13 has the potential as a biomarker. The potential of MMP-13 as a biomarker for OA also was preliminarily investigated. Ruan and his colleagues had shown that serum MMP13 level was related to some serum inflammatory factors as well as knee structural abnormalities (18). However, the suitability of MMP-13 as a biomarker for OA still is not fully understood.

Therefore, the present study aimed to evaluate the potential suitability of MMP-13 as a biomarker for OA. At first, the involvement of MMP-13 in severity-related OA progression was explored by detecting MMP-13 expression in the sever-worn cartilage and subchondral bone of the medial compartment and the less-worn cartilage and subchondral bone of the lateral compartment in patients with varus OA. Subsequently, serum MMP-13 levels were measured in patients with and without OA using enzyme-linked immunosorbent assay (ELISA) to determine the suitability of MMP-13 as a diagnostic marker. The clinical correlations between serum MMP-13 levels and the symptomatic and radiographic severity of OA were also assessed. Finally, we established the multivariable cumulative link models to evaluate the predictive potential of serum MMP-13 levels in the OA process.

## Materials and Methods

### Subjects

Patients were recruited from the Department of Orthopedics at Peking University International Hospital from January to July 2019. In total, 51 patients were enrolled in this study. Of these, 33 patients had advanced varus knee OA who underwent total knee arthroplasty (TKA) and 18 patients had meniscus injuries, ligament injuries, or patellar dislocation but with intact articular cartilages (magnetic resonance imaging). Strict standards were used to enroll patients with OA. OA was diagnosed using the diagnostic criteria of the American College of Rheumatology (19). The exclusion criteria were as follows: (1) patients with rheumatoid arthritis, systemic lupus erythematosus, ankylosing spondylitis, psoriatic arthritis, Reiter's syndrome, joint infection, and intra-articular drug injection administered 6 months before surgery; (2) patients with OA with a history of knee injuries occurred within 1 month before admission; and (3) patients who have undergone revision TKA or simultaneous bilateral TKA.

The study was approved by the Medical Science Research Ethics Committee of Peking University International Hospital (Beijing, China) (authorization number: 2018-069 [BMR]). Participants included in the study have signed informed consent forms.

### Hematoxylin and Eosin Staining and Immunohistochemical Analysis

Fifteen tibial plateau samples were obtained from patients with OA who have undergone TKA. The less-worn cartilage and subchondral bone in the lateral compartment were named as the “normal” cartilage (NC) and the “normal” subchondral bone (NS), respectively. The severe-worn cartilage and subchondral bone in the medial plateau were identified as “abnormal” cartilage (AC) and the “abnormal” subchondral bone (AS), respectively. The specimens were immediately fixed in 4% paraformaldehyde and then incubated in diethylpyrocarbonate-treated 0.2 M ethylenediaminetetraacetic acid for 8 weeks for satisfactory decalcification. Thereafter, ethanol dehydration and paraffin embedding was performed. The paraffin wax was then cut into 5-μm coronal slices. After deparaffinization, the slices were immersed in hyaluronidase (Sigma, USA) for 45 min at 37°C. Subsequently, some serial sections were stained with hematoxylin and eosin, while other sections were treated with 3% H_2_O_2_ methanol for 15 min at 25°C. After blocking with Cas Block (Invitrogen Co., Carlsbad, CA, USA), the sections were incubated with an anti-MMP-13 polyclonal antibody (1:100, Abcam Co., Cambridge, MA) overnight at 4°C. Thereafter, a polymeric secondary antibody (SuperPicTure Polymer Detection Kit, Invitrogen Co.) was used to incubate the sections. The antigen-antibody reaction was visualized using a diaminobenzidine solution, followed by counterstaining with hematoxylin. Subsequently, all sections were carried out the same differentiation procedure to clear up the bias from the incomplete hematoxylin decolorization between both the groups. Finally, the results were analyzed using the Image-Pro Plus (version 6.0, Media Cybernetics, USA). The integrated optical density (IOD), which is the sum of the absorbance of each pixel in the measured range, was assigned by the program to reflect the positive intensity of protein expression. The same program file was used for all mean IOD calculations to avoid any artificial influence in the evaluation process.

### Western Blotting

Six tibial plateau samples were obtained from patients with OA for western blotting analysis. The subchondral bone specimens were obtained from the medial and lateral tibial plateaus and then comminuted in liquid nitrogen. Approximately, 20 μg of protein was extracted from the NS and AS using ribonucleoprotein immunoprecipitation lysis buffer and then transferred onto a polyvinylidene fluoride membrane. Thereafter, the membrane was blocked with 5% non-fat dry milk and incubated with the primary antibody against MMP-13. The primary antibody was then incubated with the corresponding secondary antibodies. Thereafter, the blots were visualized by high-sensitivity enhanced chemiluminescence and quantified using Image J software.

### Measurement of Serum MMP-13 Levels

For each patient included in this study, 2.0 mL of blood sample was collected through the antecubital vein on the morning of admission. The sample was immediately centrifuged at 3,000 rpm for 10 min. Thereafter, the serum was collected and stored at −80°C for further analysis. Serum MMP-13 levels were measured using ELISA (Elabscience, China), according to the manufacturer's instructions. An automatic ELISA reader (Sunrise; Tecan, Männedorf, Switzerland) was used to measure the optical density at 450 nm.

### Clinical Associations of Serum MMP-13 Levels

The clinical associations between serum MMP-13 levels and the symptomatic and radiographic severity of OA were assessed. The Western Ontario and McMaster Universities Osteoarthritis Index (WOMAC) was used to assess the knee joint symptoms of all involved patients. The WOMAC score was obtained with a form of questionnaires, and the scores of following subgroups knee joint pain, stiffness, and physical dysfunction were self-reported.

All the patients underwent radiographic knee examination. A standing anteroposterior and lateral side view image was obtained for the symptomatic knees of every patient. The Kellgren–Lawrence (KL) grading system was applied to assess OA radiographic severity (20). The severity of OA was grouped into five grades, as reported previously. Two single trained orthopedic specialists blinded to the group assessed all radiographs.

### Establishment of Proportional Odds Logistic Regression Models

To assess the predictive value of serum MMP-13 levels in knee OA, we collected basic information and routine laboratory results for all the patients in this study. The factors involved were divided into patient-related factors and laboratory-related factors. Patient-related risk factors included age, sex, and body mass index (BMI); laboratory-related factors included serum MMP-13 level, plasma thromboplastin antecedent, prothrombin time, C-reactive protein level, neutrophil ratio, hemoglobin level, erythrocyte sedimentation rate, fibrinogen level, white blood cell count, platelet count, total protein level, alanine aminotransferase level, aspartate aminotransferase level, creatinine level, fibrin degradation product level, activated partial thromboplastin time, D-dimer level, international normalized ratio, and albumin level.

Disease diagnosis was labeled as an ordinal categorical variable with levels of 0 and 1. Level 0 represents non-OA, and level 1 is defined as OA. Two proportional odds logistic regression models were built to evaluate the extent of covariates in the prediction of knee OA. First, Model 1 integrated all factors to analyze them as potential predictors of knee OA. Model 2 then selectively assessed laboratory-related factors as predictors of knee OA. The models were assessed using the Akaike information criterion (AIC). Variable importance plots were obtained by ranking each variable according to the increase in AIC upon its removal (21). An AIC value of ≥2 represents a significantly better model (22). Odds ratios (ORs) were reported as the ratio between the 75th and 25th percentiles. *P* < 0.05 was considered to exist statistical Difference. R software (R version 3.6.3) was used to perform statistical analysis.

### Statistical Analysis

The student's *t*-test was used to compare the immunohistochemical and ELISA results. Linear regression analyses were used to examine the associations between serum MMP-13 levels (independent variable) and WOMAC. Pearson's correlation analyses was used to examine the correlation between MMP-13 levels and KL grading scores. The GraphPad Prism software (version 7.0, Inc., La Jolla, CA, USA) were used to perform all statistical analysis. *P* < 0.05 was considered to have statistical difference.

## Results

### Characterization of Subjects

**Figure 1:**
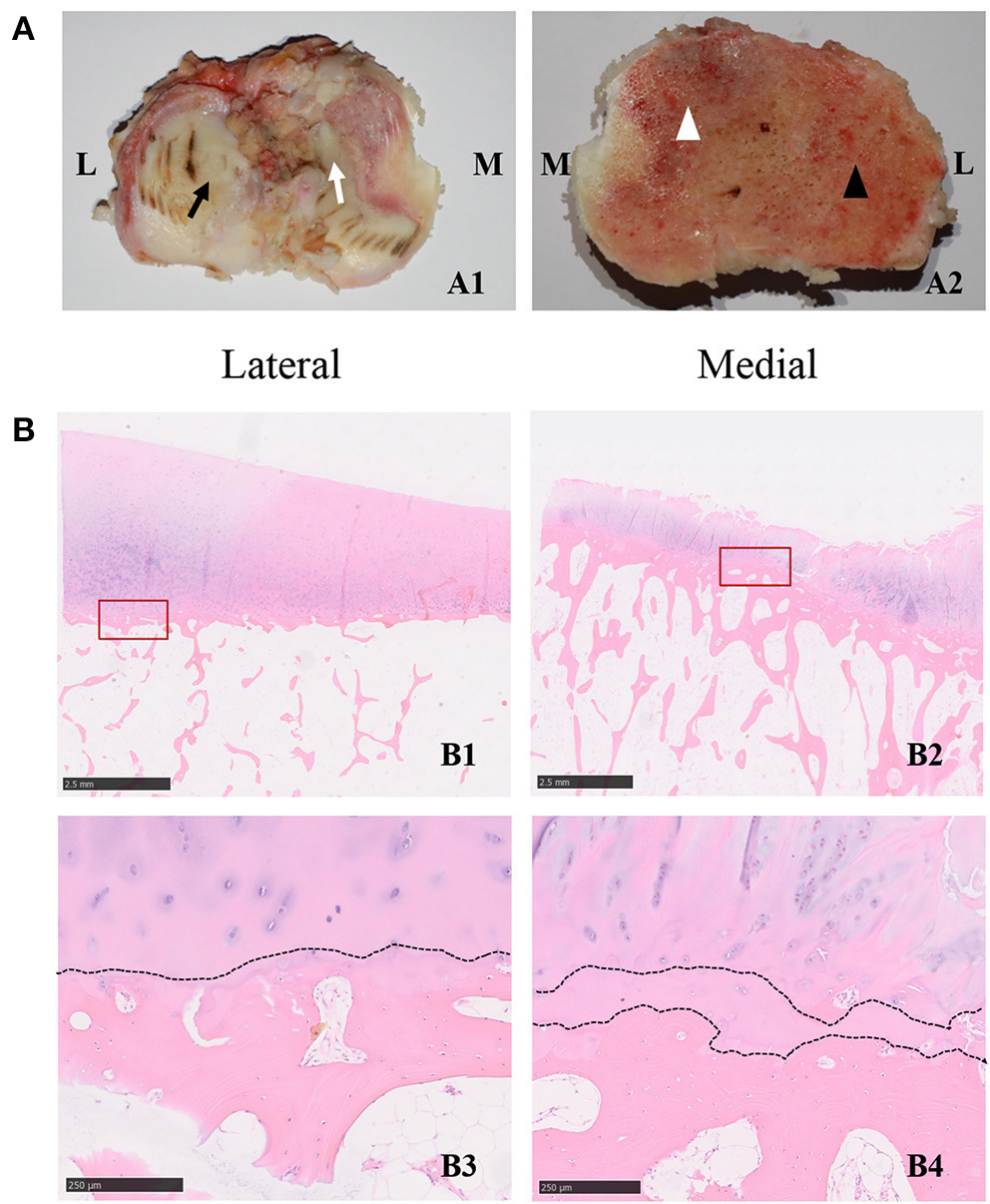
**(A,B)** Histological characterizations of the cartilage and subchondral bone in the tibial plateau of OA specimens. **(A1)** The characterizations of the cartilage in different compartments. The black arrow indicates the less-worn cartilage, while the white arrow indicates the severe-worn cartilage in the medial compartment. M indicates the medial compartment of the knee joint, and L indicates the lateral compartment of the knee joint. **(A2)** The characterizations of the subchondral bone in different compartments. The white arrowhead indicates the severe-degenerative subchondral bone, the black arrowhead indicates the mild-degenerative subchondral bone in the lateral compartment. M indicates the medial compartment of the knee joint, and L indicates the lateral compartment of the knee joint. **(B1)** Representative hematoxylin and eosin staining images of specimens in the lateral compartment of knee joint. The scale bar represents 2.50 mm. **(B2)** Representative hematoxylin and eosin staining images of specimens in the medial compartment of the knee joint. The scale bars in **B1,B2** represent 2.50 mm. The bottom two panels **(B3,B4)** are high-magnification images of the red frame in **B1** and **B2**, respectively, the dotted line indicates the tideline. The scale bar represents 250 um. The right panel **(B4)** is a high-magnification image of the red frame in **(B2)**, and the dotted lines indicate the duplicated tideline. The scale bar represents 250 um.

A total of 51 patients (74.5% woman) aged between 14 and 84 (mean: 58.4) years were enrolled in the study. There were no remarkable differences in demographic factors (sex and BMI) between patients with and without OA. Patients with and without OA were similar in terms of sex, weight, and BMI. The detailed characteristics of these patients are described in Table 1. The histological characteristics are presented in Figure 1. Sclerosis, cysts, and osteophyte formation were observed in the medial compartment of the knee joint, while the subchondral bone was relatively normal in the lateral compartment. As shown by histological staining, duplicated tideline was observed in the medial compartment. In addition, the subchondral bone in the medial compartment had more trabecular bone than the subchondral bone in the lateral compartment. These findings suggest that different compartments might undergo different stages of the disease.

**Table 1:** Patient characteristics in both the groups.

	**Group** ^**OA**^ **(*n* = 37)**	**Group** ^**NO-OA**^ **(*n* = 18)**	***P*-value**
	**Mean ± SD**	**Mean ± SD**	
Age (years)	68.4 ± 6.25	39.6 ± 18.00	0.000
Female/male	31/6	10/8	0.10
Height (cm)	162.24 ± 6.68	174.00 ± 12.18	0.001
Weight (kg)	67.48 ± 13.99	77.38 ± 16.83	0.11
BMI (kg/m^2^)	25.72 ± 5.43	25.4 ± 3.70	0.88
Serum MMP-13 level (ng/ml)	5.87 ± 3.31	5.38 ± 3.87	0.73

### MMP-13 Expression in Different Compartments of Patients With OA

**Figure 2:**
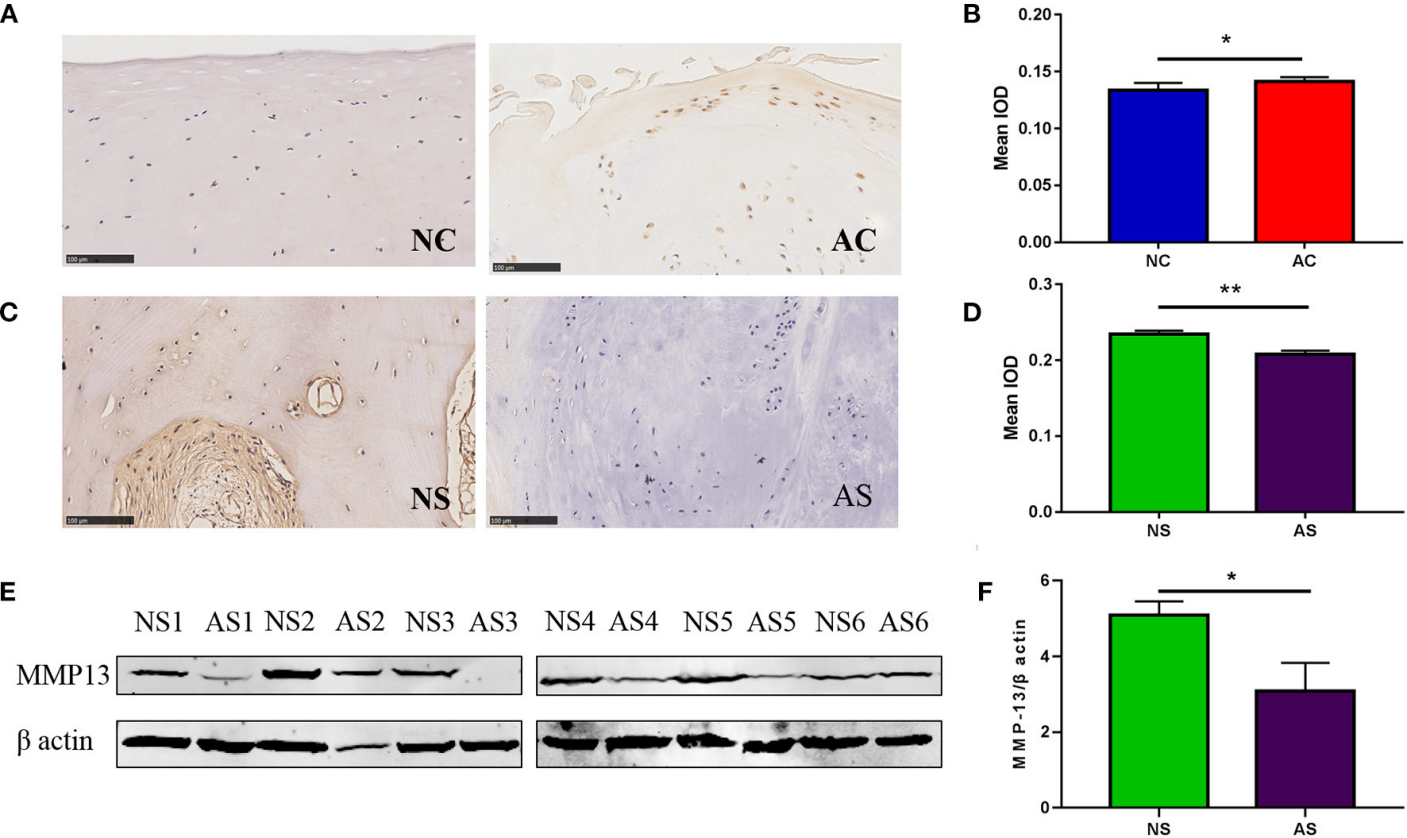
MMP-13 expression in the cartilage and subchondral bone of patients with OA. The representative images of MMP-13 levels in the NC and AC **(A)** and the semiquantitative results of MMP-13 expression in the NC and AC **(B)**. NC represents less-worn cartilage, AC represents severe-worn cartilage; IOD indicates integrated optical density (*p* < 0.05). The error bars indicate mean ± standard error of the mean. *p < 0.05. **(C)** The representative images of MMP-13 levels in the subchondral bone. NS represents less-worn subchondral bone, AS represents severe-worn subchondral bone. **(D)** The semiquantitative results of MMP-13 expression in the subchondral bone in the NS and AS. NS represents less-worn subchondral bone, AS represents severe-worn subchondral bone; IOD indicates integrated optical density. The error bars indicate mean ± standard error of the mean. *p < 0.05, **p < 0.01. **(E)** Western blotting findings of MMP-13 expression in the subchondral bone. NS represents less-worn subchondral bone, AS represents severe-worn subchondral bone. **(F)** The quantification of western blot shows the expression of MMP-13 in subchondral bone. NS represents less-worn subchondral bone, AS represents severe-worn subchondral bone. The error bars indicate mean ± standard error of the mean. **p < 0.01.

The immunohistochemistry results are shown in Figures 2A–D. MMP-13-positive cells were scarce in the less-worn cartilage of the lateral plateau, while they were abundant in the medial plateau. However, there were more MMP-13-positive cells in the subchondral bone of the lateral plateau than in the medial plateau. The semiquantitative results confirmed that the MMP-13 expression level was significantly higher in the severe-worn cartilage in the medial tibial plateau than in the less-worn cartilage in the lateral plateau. However, in the subchondral bone, the expression of MMP-13 were significantly higher in the less-worn lateral tibial plateau than in the severe-worn medial plateau, which also was demonstrated by the results of western blotting.

### Serum MMP-13 Level

**Figure 3:**
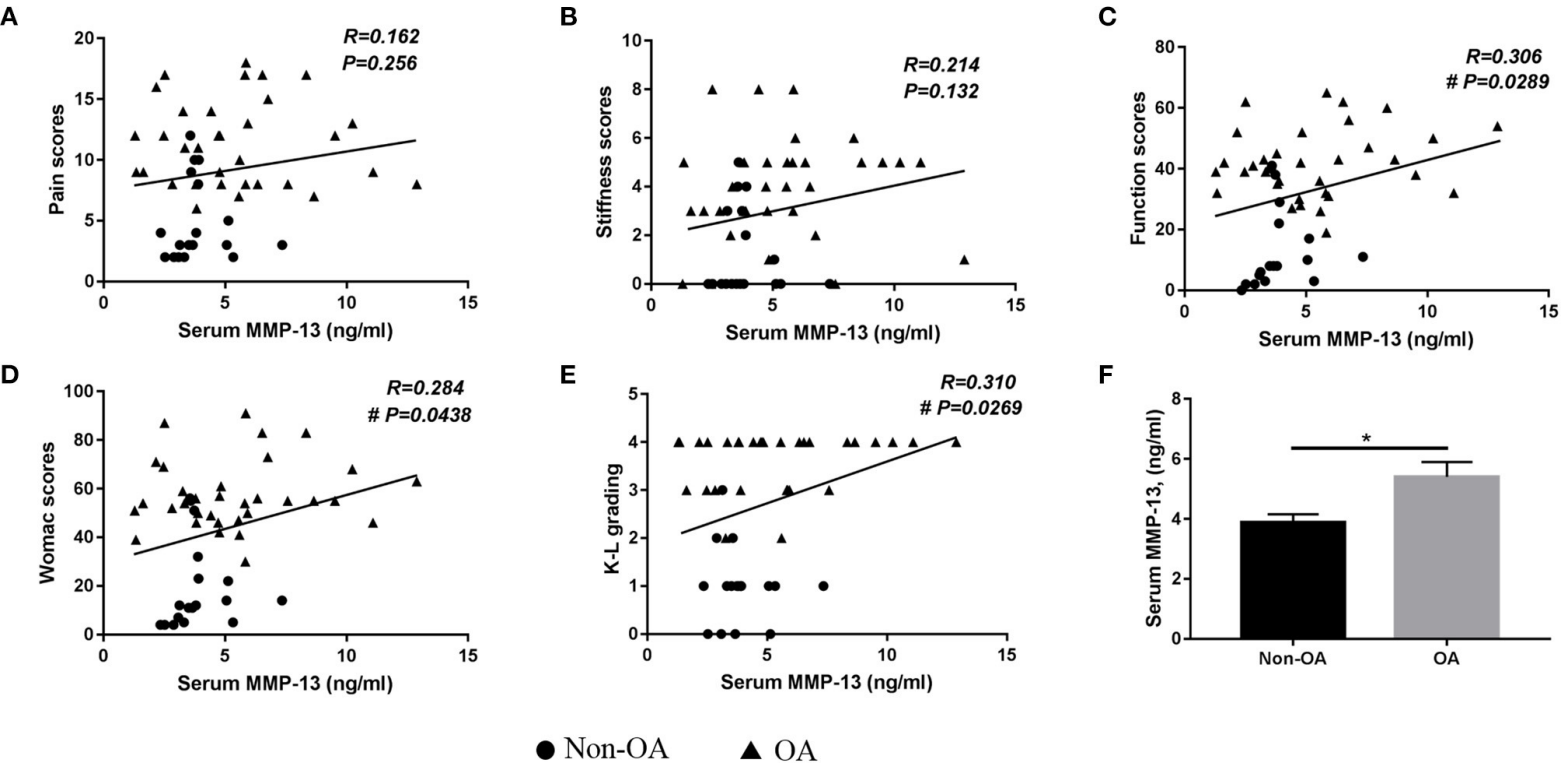
Analysis of factors relating serum MMP-13 with osteoarthritic symptoms and radiographic severity. Correlations between plasma MMP-13 levels and **(A)** pain scores, **(B)** stiffness scores, **(C)** function scores, **(D)** total Western Ontario and McMaster Universities Osteoarthritis Index scores, **(E)** Kellgren–Lawrence grading in all patients, **(F)** serum MMP-13 levels in patients with and without OA. Value = mean ± standard error of the mean. OA, osteoarthritis. *p < 0.05; ^#^indicates the difference was statistically significant (*p* < 0.05).

The ELISA results are shown in Figure 3F. There were significantly increased serum MMP-13 levels in patients with OA than in those without OA.

### Association Between Serum MMP-13 Level and WOMAC Scores

A remarkable correlation was found between serum MMP-13 levels and total WOMAC scores (*r* = 0.284, *p* = 0.0438) (Figures 3A–D). The pain [*r* = 0.162, *p* = 0.256] and stiffness [*r* = 0.214, *p* = 0.132], two subgroups of WOMAC grading system, showed no association with serum MMP-13 levels, while function scores were significantly related to serum MMP-13 levels (*r* = 0.306, *p* = 0.0289).

### Association Between Serum MMP-13 Level and K-L Grading Scores

The results of the association between serum MMP-13 levels and radiographic severity of OA are presented in Figure 3E. There was a significant association between serum MMP-13 levels and KL grading scores (*r* = 0.310, *p* = 0.0269).

### Predictive Value of Serum MMP-13 Levels for OA

**Figure 4:**
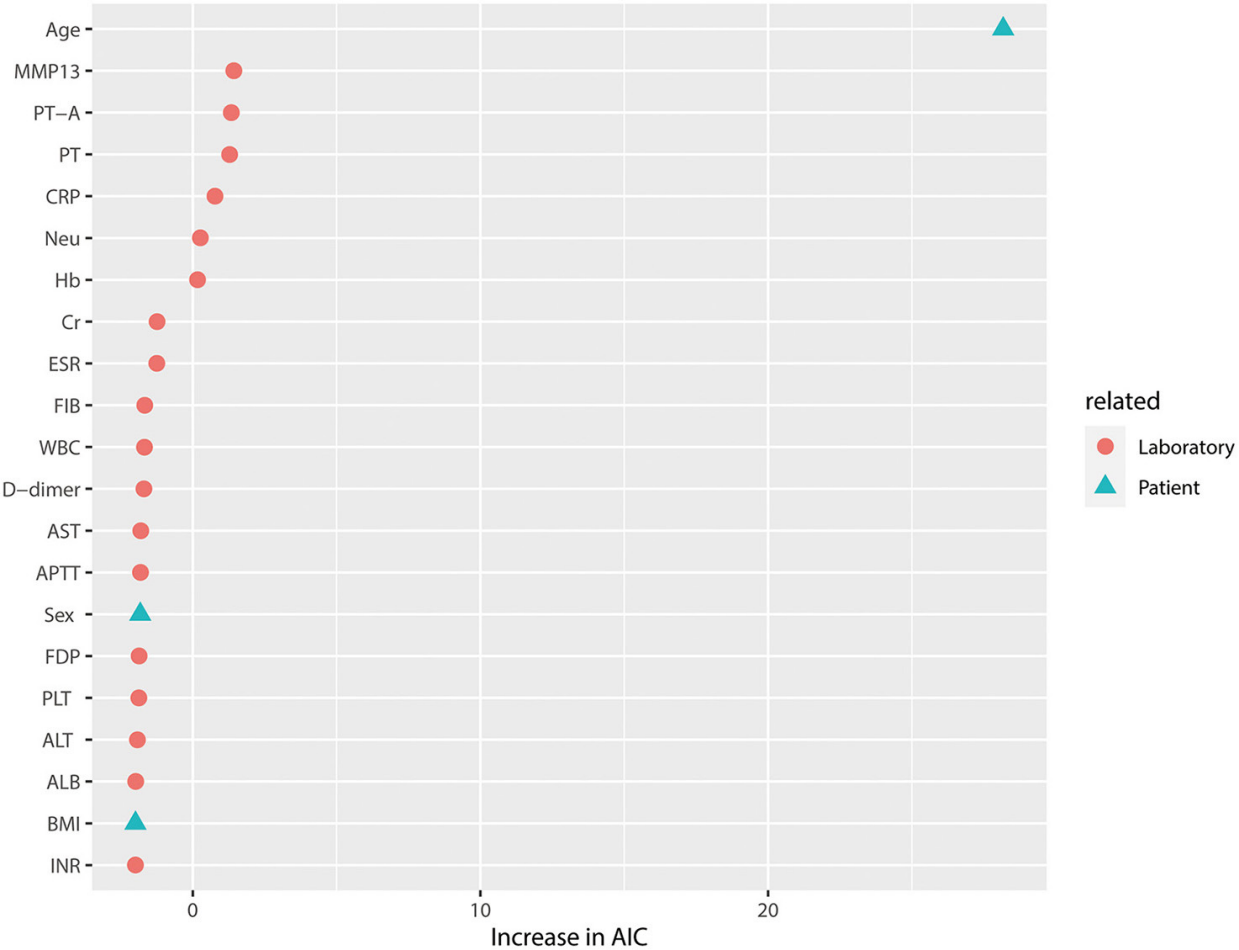
Relative variable importance of each predictor in the full model of OA grouped by risk factors related to laboratory and patient. The factors for knee OA were involved in the full model. Patient age was the major risk factor for knee OA in the full model. Despite the AIC value of <2, MMP-13 was also a major risk factor for knee OA compared with other laboratory-related factors. ESR, erythrocyte sedimentation rate; PT-A, plasma thromboplastin antecedent; PT, prothrombin time; CRP, C-reactive protein; Neu%, neutrophil ratio; Hb, hemoglobin; Cr, creatinine; ESR, erythrocyte sedimentation rate; FIB, fibrinogen; WBC, white blood cell count; AST, aspartate aminotransferase; APTT, activated partial thromboplastin time; FDP, fibrin degradation product; PLT, platelet count; ALT, alanine aminotransferase; ALB, albumin level; INR, international normalized ratios; BMI, body mass index.

The predictive value of serum MMP-13 levels and several other risk factors that are potentially associated with OA was evaluated. The results are shown in Figure 4 and Table 2. In model 1, patient age seemed to be a major risk factor for knee OA (*p* < 0.001). After excluding patient-related factors, serum MMP-13 level seems to be the best laboratory-related factor in predicting knee OA (*p* < 0.05). As shown in model 2, platelet count was also associated with knee OA despite the low AIC value (*p* < 0.05).

**Table 2:** Predictive factors of knee osteoarthritis for model 1 (patient-and laboratory-related factors) and model 2 (laboratory-related factors).

**Variable**	**Model 1**		**Model 2**	
	**OR (95% CI)**	***P-*value**	**OR (95%CI)**	***P*-value**
**Patient-related risk factors**				
Age	1.02 (1.01–1.03)	<0.001**		
**Sex**				
Female	1.06 (0.73–1.56)	0.755		
Male	—	—		
BMI	1.00 (0.98–1.02)	0.950		
**Laboratory-related risk actors**				
MMP-13	1.03 (0.99–1.08)	0.166	1.07 (1.01–1.13)	0.037*
CRP (mg/L)	1.02 (0.99–1.05)	0.214	1.03 (0.99–1.07)	0.207
ESR (mm/H)	0.99 (0.98–1.01)	0.518	0.99 (0.97–1.01)	0.347
WBC	1.02 (0.94–1.11)	0.672	1.01 (0.90–1.13)	0.884
Neu (%)	0.99 (0.98–1.00)	0.132	0.98 (0.97–1.00)	0.061
Hb	0.99 (0.98–1.01)	0.271	1.00 (0.98–1.01)	0.572
PLT	1.00 (0.99–1.00)	0.789	1.00 (0.99–1.00)	0.041*
Alb (g/L)	1.00 (0.98–1.02)	0.925	1.00 (0.97–1.03)	0.895
ALT (U/L)	1.00 (0.98–1.02)	0.840	0.99 (0.96–1.01)	0.308
AST (U/L)	1.01 (0.97–1.04)	0.743	1.03 (0.98–1.08)	0.252
Cr (mmol/L)	1.00 (1.00–1.00)	0.515	1.00 (1.00–1.00)	0.463
PT(S)	0.35 (0.08–1.54)	0.176	0.84 (0.14–5.09)	0.854
PT-A	0.92 (0.81–1.03)	0.172	0.99 (0.85–1.15)	0.865
INR	1.00 (0.93–1.07)	0.950	1.01 (0.92–1.11)	0.801
APTT (S)	1.01 (0.96–1.05)	0.751	0.99 (0.93–1.05)	0.724
FIB	1.00 (1.00–1.00)	0.665	1.00 (1.00–1.01)	0.222
FDP	0.98 (0.84–1.14)	0.783	0.94 (0.76–1.15)	0.528
D-dimer	1.00 (1.00–1.00)	0.681	1.00 (1.00–1.00)	0.474

*Model 1: patient- and laboratory-related factors; Model 2: laboratory-related factors.*

*p < 0.05, **p < 0.01. Body mass index (BMI), C-reactive protein (CRP), erythrocyte sedimentation rate (ESR), white blood cell count (WBC), neutrophil ratio (Neu%), hemoglobin (Hb), platelet count (PLT), total protein (TP), albumin level (ALB), alanine aminotransferase (ALT), aspartate aminotransferase (AST), creatinine (Cr), international normalized ratios (INR), fibrin degradation product (FDP), activated partial thromboplastin time (APTT), D-dimer, fibrinogen (Fib), prothrombin time (PT), and plasma thromboplastin antecedent (PT-A).

## Discussion

Although the involvement of MMP-13 has been suggested in the pathogenesis of OA (13), few clinical studies have examined its potential value as a biomarker in diagnosing, assessing severity, and predicting the risk of OA. In the present study, we investigated the potential suitability of MMP-13 as a biomarker for knee OA at local to systemic levels. We found that the local MMP-13 level in the severe-worn cartilage in the medial tibial plateau was significantly higher than that in the less-worn cartilage in the lateral plateau, but MMP-13 expression in the subchondral bone showed an opposite trend in knee OA. Moreover, the serum MMP-13 levels were found to be positively associated with the symptoms and radiographic severity of OA. Furthermore, serum MMP-13 levels showed a good predictive value for OA. These findings suggest that serum MMP-13 level has a potential possibility to serve as a biomarker for knee OA. However, the clinical applicability desires further investigation.

In this study, the patients involved in the control group were those who had meniscus injuries, ligament injuries, and all were not in line with the diagnostic criteria of the American College of Rheumatology at the present stage. Moreover, they all were acute injuries. A previous study showed that patients with the anterior cruciate ligament injuries displayed the first radiologic signs of OA on average about 10 years after the injury. For patients who had an isolated meniscus injury, the average time until development of radiologic signs of OA was about 5–15 years (23). Therefore, the patients included in control group have a short course of the disease and at present did not develop OA, which could be considered as a good control with these patients have developed OA.

In the present study, the relationship between local MMP-13 levels and disease severity in the progression of OA was confirmed. In clinical situations, predominantly torn cartilage is more prevalent in the medial compartment than in the lateral compartment of patients with OA. These differences in several compartments might reflect the various stages of the disease, as indicated by the histological findings of our study. Therefore, evaluation of MMP-13 levels in different compartments was helpful in determining its value in terms of varying disease severities. However, only a few studies have reported MMP-13 expression in different compartments of the human knee joints. Herein, we found that MMP-13 expression was higher in the sever-worn cartilage of the medial compartment than in the less-worn cartilage of the lateral compartment in patients with OA. This result was in line with a previous study that showed that collagenase activity was higher in OA cartilage than in normal cartilage (24). Interestingly, MMP-13 expression in the subchondral bone showed an opposite trend. This is consistent with the finding of a study conducted by Mazur et al. who reported that MMP-13 was downregulated in the subchondral bone at end-stage knee OA. Here, we postulated two reasons for this discordance in MMP-13 levels between the cartilage and subchondral bone. First, reduction in MMP-13 activity and suppression of perilacunar remodeling have reportedly been in the subchondral bone in advanced knee OA (25–27). These changes lead to collagen disorganization and hyper-mineralization, which are also major histological features of the sclerotic subchondral bone at the later stage of knee OA. The functionality and integrity of the articular cartilage depend on the mechanical and nutritional support of the subchondral bone (28, 29). Degeneration of the subchondral bone disrupts the metabolic homeostasis of the cartilage, which is characterized by the increased expression of MMP-13 and the disturbance in the extracellular matrix in the articular cartilage. Additionally, as found in our study, the duplicated tideline was present in the medial tibial plateau, which accords with a previous findings (30). The duplicated tideline might block the exchange of cytokines, including MMP-13, between the cartilage and the subchondral bone. This results in the differential expression of MMP-13 in the cartilage and the subchondral bone along the tibial plateau. Although the exact reasons for this discordance between the cartilage and subchondral bone remain unclear, the different MMP-13 levels throughout the compartments indicate that it is related to disease severity.

Differential expression of MMP-13 suggests its role in the progression of OA and its suitability in distinguishing disease severity. However, the clinical application value of local MMP-13 levels as an OA biomarker is limited. Therefore, the systemic level of serum MMP-13 was further investigated to determine whether it could aid in the diagnosis of OA. Our results showed that serum MMP-13 levels were significantly higher in patients with advanced OA compared with patients without OA, which, to some extent, suggests the diagnostic value of serum MMP-13 levels for the disease.

To further determine the value of serum MMP-13 in favor of distinguishing the clinical severity of OA, the associations between MMP-13 and the symptomatic and radiographic characteristics of patients with advanced OA were explored. The pain, stiffness, and physical dysfunction are the most common symptoms in patients with knee OA, which seriously affect their quality of life (31). The WOMAC is the most commonly used disease-specific measure for outcomes of OA. This system comprised 24 items with a total score and the following three subscales: pain, stiffness, and physical function. The current results demonstrated that serum MMP-13 was significantly associated with the total WOMAC scores and function scores but was not associated with the other two subscales (pain, stiffness). These results suggest that MMP-13 has a better association with symptomatic OA. This might provide important clues for developing symptom-modifying strategies for knee OA. For example, an elderly patient complaining of knee pain was diagnosed with a knee ligament injury and knee OA. However, radiographic findings were not sufficient to diagnose patients with severe knee OA. At such a time, the serum MMP-13 could allow for further judgment of the patient's condition. The higher the serum MMP-13 level, the better the osteoarthritic contribution to clinical symptoms. When the serum MMP 13 level increased to a certain level, knee OA should be considered as the “culprit” of osteoarthritic symptoms. Thus, symptoms-modifying strategies should be inclined toward OA. However, this hypothesis needs to be confirmed in future clinical trials.

Although MMP-13 is well associated with OA symptoms, there is a widespread belief that symptomatic knee OA exists a high discordance with radiographic OA (32). Therefore, we assessed the relationship between MMP-13 levels and the radiographic severity of OA. In our study, MMP-13 was found to be significantly associated with the K-L grading scores, which is in agreement with the findings of a previous study (18). Therefore, serum MMP-13 levels can reflect both the symptomatic and the radiographic severity of OA, suggesting that MMP-13 has a potential value as a biomarker for knee OA.

In the present study, the predictive value of MMP-13 as a risk factor was determined by multivariable cumulative link models. Our results showed that serum MMP-13 could serve as a valuable risk factor for knee OA. This is consistent with the findings of a previous research that showed that MMP-13 can serve as an indicator for joint degeneration (18). Moreover, age and platelet count were also found to have a remarkable value for predicting OA. This result was consistent with a previous study reported that platelets were an indicator of the severity of hip OA (33). More recently, it has been found that age plays an important role in OA development. Age-associated oxidative stress and related mitochondrial dysfunction might lead to senescence in joint tissue cells (34). Thus, it is not surprising that age has the perfect predictive value of OA.

There are several limitations in our study. First, the sample size in our study was relatively small. It is possible that more significant associations can be found in future studies when larger sample size is used. Second, the patients with OA in this study were at an advanced stage of the disease. Thus, the predictive value of serum MMP-13 in the early-stage OA remains unclear. Future longitudinal studies, especially involving the early-stage OA, are required to verify these preliminary findings.

In conclusion, our results showed that MMP-13 is valuable for diagnosing, assessing clinical severity, and predicting OA in the end-stage period of the disease, which suggests MMP-13 has potential possibility as a biomarker for knee OA. However, the underlying mechanisms and the clinical application require to be further investigated.

## Data Availability Statement

The original contributions presented in the study are included in the article/supplementary material, further inquiries can be directed to the corresponding author/s.

## Ethics Statement

The studies involving human participants were reviewed and approved by Peking University International Hospital Medical Science Research Ethics Committee. Written informed consent to participate in this study was provided by the participants' legal guardian/next of kin.

## Author Contributions

TZ, YT, and YL formulated the study hypothesis. QT, XX, and FL designed the study. ZC, KZ, FL, FZ, BY, and ZX provided the study subjects. TZ, QT, and XX collected the clinical date and performed the experiment, analyze the date and drafted the manuscript. YT, YL, and TZ review the manuscript. All authors contributed to the article and approved the submitted version.

## Funding

This work was supported by the grants from the AO Trauma Asia Pacific under AO Foundation (AOTAP20-53) and National Natural Science Foundation of China (82002030).

## Conflict of Interest

The authors declare that the research was conducted in the absence of any commercial or financial relationships that could be construed as a potential conflict of interest.

## Publisher's Note

All claims expressed in this article are solely those of the authors and do not necessarily represent those of their affiliated organizations, or those of the publisher, the editors and the reviewers. Any product that may be evaluated in this article, or claim that may be made by its manufacturer, is not guaranteed or endorsed by the publisher.

## Abbreviations

AIC: Akaike information criterion
BMI: Body mass index
CRP: C-reactive protein
ELISA: Enzyme-linked immunosorbent assay
ESR: Erythrocyte sedimentation rate
FDP: Fibrin degradation product
INR: International normalized ratios
IOD: Integrated optical density
OR: Odds ratios
PT: Prothrombin time
TKA: Total knee arthroplasty
TP: Total protein
WBC: White blood cell count.
